# Exosomes secreted by mesenchymal stromal/stem cell-derived adipocytes promote breast cancer cell growth via activation of Hippo signaling pathway

**DOI:** 10.1186/s13287-019-1220-2

**Published:** 2019-04-11

**Authors:** Shihua Wang, Xiaodong Su, Meiqian Xu, Xian Xiao, Xiaoxia Li, Hongling Li, Armand Keating, Robert Chunhua Zhao

**Affiliations:** 1 Institute of Basic Medical Sciences Chinese Academy of Medical Sciences, School of Basic Medicine Peking Union Medical College, Center of Excellence in Tissue Engineering Chinese Academy of Medical Sciences, Beijing, 100005 China; 2 Brain Tumor Research Center, Beijing Neurosurgical Institute, Beijing Tiantan Hospital affiliated to Capital Medical University, Tiantan Xili 6, Dongcheng District, Beijing, 100050 China; 3 Department of Genetics and Cell Biology, Basic Medical College, Qingdao University, 308 Ningxia Road, Qingdao, 266071 China; 4 Cell Therapy Program, Princess Margaret Hospital, Toronto, Ontario M5G 2M9 Canada; 5 Institute of Biomaterials and Biomedical Engineering, University of Toronto, Toronto, Ontario M5G 2M9 Canada; 6 Institute of Medical Science, University of Toronto, Toronto, Ontario M5G 2M9 Canada

**Keywords:** Exosomes, Adipocyte, Mesenchymal stromal/stem cell, Breast cancer, Hippo signaling pathway

## Abstract

**Objective:**

Although adipocytes are the most abundant stromal cell component in breast cancer tissues, their interaction with breast cancer cells has been less investigated compared to cancer-associated fibroblasts or macrophages. Exosomes are a novel way of cell-cell communication and have been demonstrated to play an important role in various biological processes. However, to our knowledge, only a few studies have reported the effects of adipocyte exosomes on tumor development. Here, utilizing exosomes isolated from in vitro mesenchymal stromal/stem cell (MSC)-differentiated adipocytes, we systematically investigated this issue in a breast cancer model.

**Material and methods:**

Exosomes were isolated from MSC-differentiated adipocytes and added to breast cancer cells MCF7. Cell proliferation was detected by MTS, and migration was analyzed by wound healing and transwell assay. An in vivo mouse xenograft model was used to evaluate MSC-differentiated adipocyte exosomes’ contribution to tumor growth. Signaling pathway activation was evaluated by western blot and immunofluorescence staining.

**Results:**

We found MSC-differentiated adipocyte-derived exosomes are actively incorporated by breast cancer cell MCF7 and subsequently promote MCF7 proliferation and migration as well as protect MCF7 from serum derivation or chemotherapeutic drug-induced apoptosis in vitro. In the in vivo mouse xenograft model, depletion of exosomes reduces tumor-promoting effects of adipocytes. Transcriptomic analysis of MSC-differentiated adipocyte exosome-treated MCF7 identified several activated signaling pathways, among which we confirm the Hippo signaling pathway and found a blockade of this pathway leads to a reduced growth-promoting effect of adipocyte exosomes.

**Conclusion:**

Taken together, our findings provide new insights into the role of adipocyte exosomes in the tumor microenvironment.

## Background

Breast cancer is one of the most common cancers and the second leading cause of cancer-related mortality in women worldwide [1]. Various factors such as genetic and epigenetic mutations, abnormal hormone levels, and environmental stimulus contribute to breast cancer development [2, 3]. Emerging evidence indicates that the tumor microenvironment plays a vital role in cancer initiation and progression [4]. The tumor microenvironment consists of a number of different components including non-malignant cells, surrounding blood vessels, extracellular matrix (ECM), and signaling molecules [5]. In breast cancer, studies focusing on interactions between cancer cells and tumor microenvironment have emphasized the important roles of stromal compartments such as cancer-associated fibroblasts and cancer-associated macrophages [6]. One critically important, yet often overlooked, tumor microenvironment component is adipocytes, which are the most abundant stromal cells in human breast tissue. Increasing evidence suggests that adipocytes are not merely an energy-storing cell, but can function as endocrine cells by producing hormones, growth factors, cytokines, and adipokines [7, 8]. Herroon et al. found bone-trophic breast tumor cells could upregulate the oxidative stress enzyme upon exposure to adipocyte-rich environments in vitro or in vivo [9]. Similarly, a conditioned medium from adipocytes was reported to increase motility of breast cancer cell lines [10]. Adipocytes could transfer free fatty acids (FFAs) to stimulate breast cancer invasion via metabolic remodeling of tumor cells [11]. These studies suggest there is an intimate interaction between breast cancer cells and adipocytes. However, the underlying mechanism governing adipocyte crosstalk with breast cancer cells is not fully understood.

Exosomes are a novel way of cell-cell communication and play an important role in tumor development [12–14]. Adipocyte-secreted exosomes have been shown to aggravate atherosclerosis by increasing angiogenesis [15] and induce insulin resistance in skeletal muscle through repression of PPARγ [16]. Adipocytes, which are specialized in storing and releasing FFAs, are able to shift tumor metabolism toward the use of FFAs via extracellular vesicles [17]. Currently, most studies use mouse cell line 3T3-L1-differentiated adipocytes as a cellular model. Here, we induced human adipose tissue-derived mesenchymal stromal/stem cells (MSCs) into adipocytes. MSCs were defined in 2006 by the International Society of Cellular Therapy (ISCT) as cells with the three properties: (1) be adherent to plastic under standard tissue culture conditions, (2) express certain cell surface markers such as CD73, CD90, and CD105 and lack expression of other markers including CD45, CD34, CD14, or CD11b, CD79alpha, or CD19 and HLA-DR surface molecules, (3) have the capacity to differentiate into osteoblasts, adipocytes, and chondroblasts under in vitro conditions [18]. According to ISCT criteria, the isolated MSCs are a heterogeneous population of cells containing both stem cells and cells with lower multipotential properties [19]. So many experts recommend the use of mesenchymal stromal/stem cells (MSCs) [20–22]. MSCs, especially adipose tissue-derived MSCs, can be differentiated into adipocytes under proper in vitro culture conditions [23, 24]. To our knowledge, only a few studies have reported the effects of adipocyte exosomes on tumor development. Here, utilizing exosomes isolated from in vitro MSC-differentiated adipocytes, we systematically investigated this issue in breast cancer. We found mesenchymal stem cell (MSC)-differentiated adipocyte exosomes could promote breast cancer cell proliferation and migration as well as protect breast cancer cells from serum derivation or chemotherapeutic drug-induced apoptosis in vitro. Furthermore, exosomes contribute to in vivo tumor growth in a mouse xenograft model. Mechanistically, the Hippo signaling pathway was demonstrated to be partially responsible for the tumor-promoting effects of MSC-differentiated adipocyte exosomes. Taken together, our findings provide new insights into the role of adipocyte exosomes in the tumor microenvironment.

## Material and methods

### Cell line and culture

MCF-7 cells were purchased from The Cell Center of the Chinese Academy of Medical Sciences (Beijing, China) and cultured in Dulbecco’s modified Eagle’s medium with 4.5 g/L glucose (H-DMEM) containing 10% FBS, 100 U/ml penicillin, and 100 μg/ml streptomycin.

### Isolation and expansion of MSCs from adult human adipose tissue

Human adipose tissue was obtained from donors undergoing liposuction according to procedures approved by the Ethics Committee at the Chinese Academy of Medical Sciences and Peking Union Medical College. The isolation and culture procedure for human adipose tissue-derived MSCs was described in our previous papers [25, 26]. MSCs of the third passage were used for the following study. It has been reported that senescent cells may produce anti-cancer factors that block cancer growth [27]. We performed beta-galactosidase assay and found no senescent cells at passage 3.

### Adipogenesis and analysis

The culture-expanded cells of the third passage at 100% confluence were induced in the following adipogenic media for 12 days: H-DMEM supplemented with 10% FBS, 1 μM dexamethasone, 0.5 mM isobutylmethylxanthine, and 1 mM ascorbic acid (all reagents were from Sigma Aldrich). For cell staining, cells were stained with filtered Oil Red O solution (stock solution: 3 mg/ml in isopropanol; working solution: 60% Oil Red O stock solution and 40% distilled water) for 1 h at room temperature and 2 μM BODIPY staining solution for 15 min at 37 °C, respectively. Then, cells were washed with water to remove unbound dye, visualized by microscopy, and photographed.

### Exosome isolation and analysis

Cell cultures used to isolate exosomes were grown in serum-free H-DMEM. Exosomes were isolated from conditioned media collected at 48 h by serial centrifugation as previously described (Thery et al., 2006: Isolation and Characterization of Exosomes from Cell Culture Supernatants and Biological Fluids), and exosome pellets were resuspended in PBS. Exosomes were quantified by BCA protein quantification. Morphology of the exosomes was examined by electron microscopy using negative staining. To examine exosome markers, cellular and exosome protein was extracted by 10% SDS lysis. Exosome markers include CD63 (Proteintech), TSG101 (Proteintech), Calnexin (Proteintech), and beta-actin (Santa Cruz). Exosome sizes were identified by nanoparticle tracking analysis with ZETA VIEW (Particle Metrix), and the exosomes were diluted 100–400 times in 100 μL of sterile PBS. The exosome-depleted culture media were obtained after exosome isolation from conditioned culture media by ultracentrifugation. Exosomes taking-up was investigated by labeling exosomes with DiI (Invitrogen) and labeling cell nucleus with Hochest33342. Dye transfer was visualized by fluorescent microscopy. The co-culture of exosome pellets and MCF-7 cells were performed at a concentration of 200 μg/ml.

### Proliferation assay

Cells were plated in 96-well plates at a density of 2 × 103 cells per well. To reduce differences within the group, each group of cells samples a set of five parallel holes. Then, the cells were incubated with an MTS reagent (CellTiter 96® AQueous One Solution Cell Proliferation Assay, Promega) for 2 h in 37 °C and 5% CO_2_. The optical density (OD) value was measured by an ELISA reader (Bio-Tek).

### Scratch assay

1 × 10^6^ cells/well were plated in a 24-well plate. The other day, the cells formed a monolayer. A 10-μL pipette tip was used to make a straight scratch. The culture medium was changed to H-DMEM which contained exosomes or PBS in the same volume. The cells were incubated in a 37 °C humidified incubator with 5% CO_2_. The wound distance was measured in a light microscope and the total time was 24 h. All samples were tested in triplicate, and the data are expressed as the mean ± SD.

### Migration assay

Briefly, MCF-7 cells were cultured in H-DMEM which contained exosomes or PBS in the same volume for 48 h. Then, 200 μL of cells (1 × 10^6^/ml) suspended in a DMEM-only medium was loaded in triplicate upper chambers of the transwell chambers (Costar) with a 8-μm pore size. And a 600-μL H-DMEM medium with 20% FBS was added into the lower chamber. After incubated for 12 h in a 37 °C humidified incubator with 5% CO_2_, the migrated cells were fixed, washed, and stained with crystal violet staining solution. Then the staining solution was extracted by 30% glacial acetic acid, and the optical density (OD) value was measured by an ELISA reader (Bio-Tek). All samples were tested in triplicate, and the data are expressed as the mean ± SD.

### Apoptosis analysis by flow cytometry

Annexin V-FITC/PI double labeling was used to determine the apoptosis of MCF-7 cells cultured in H-DMEM which contained exosomes or PBS in the same volume, as well as the apoptosis-inducing effect of 60 μM 5FU (MEC) on MCF-7 cells cultured in H-DMEM which contained 10% FBS with exosomes or PBS in the same volume. Cells were plated in the 12-well plate and treated the same as above. After 48 h treatment, the cells were harvested by trypsinization and incubated with FITC-conjugated Annexin V and PI according to the manufacturer’s instruction (BD Biosciences). The flow cytometer BD Bioscience Accuri C6 and ModiFit Software were applied for apoptosis analysis. A total of at least 1 × 104 cells were analyzed for each sample. All samples were tested in triplicate, and the data are expressed as the mean ± SD.

### Animal experiments

BALB/C mice (5–6 weeks) were purchased from the Laboratory Animal Center of the Chinese Academy of Medical Sciences (Beijing, China). All mice were bred and maintained under specific pathogen-free conditions. Animal use and experimental procedures were approved by the Animal Care and Use Committee of the Chinese Academy of Medical Sciences. The mice were divided into four groups: one group received a subcutaneous injection of 2 × 10^6^ MCF-7 cells. The second group received an injection of 2 × 10^6^ MCF-7 cells and 1 × 10^6^ MSC-differentiated adipocytes. The other group received an injection of 2 × 10^6^ MCF-7 cells and 1 × 10^6^ MSC-differentiated adipocytes pretreated with 20 μM GW4869 for 48 h. When MSC-differentiated adipocytes were pretreated with 20 μM GW4869 for 48 h, the isolated adipocyte exosomes were undetectable while, in the control group, exosome yield is about 100 μg/10^7^ MSC-differentiated adipocytes. The last group received 2 × 106 MCF-7 cells and 20 μM GW4869. The tumor volume was measured weekly. The tumor tissues were fixed with 10% PFA. Each group was treated with HE and Ki67 staining. Ki67 antibody was purchased from Proteintech.

### Quantitative real-time polymerase chain reactions

Cultured cells were lysed by TRIzol (Invitrogen), and RNA was extracted according to the manufacturer’s instruction. One microgram of total RNA from each sample was reverse transcribed using M-MLV (Takara) in a final volume of 30 μL. The polymerase chain reaction (PCR) amplification was carried out using the Step-one System (Bio-Rad) with SYBR Green Mastermix (Takara). All quantitative real-time PCR (qRT-PCR) results were carried out in duplicate and normalized to GAPDH.

### Western blotting

After washing twice with cold PBS, cells were lysed in RIPA lysis buffer (Beyotime) with 1 mM PMSF and protease inhibitor cocktail on ice for 30 min, manually scraped from culture plates, and then quantified using the BCA Protein Assay Kit (Beyotime). Proteins were separated on 10% sodium dodecyl sulfate–polyacrylamide gel electrophoresis (SDS-PAGE) gels, electroblotted onto a polyvinylidene difluoride (PVDF) membrane (0.22 μm, Millipore). The membranes were blocked with 5% BSA and incubated with specific antibodies overnight at 4 °C and then were incubated with horseradish peroxidase (HRP)-conjugated secondary antibody for 1 h at room temperature. The primary antibodies were as follows: GAPDH (Santa Cruz), YAP, p-YAP (Ser127), JAK2, p-JAK2 (Tyr1007), Stat3, p-Stat3 (Tyr705), SAPK/JNK, p-SAPK/JNK (Thr183/Tyr-185), P38, p-P38 (Thr180/Tyr182), ERK, p-EKR1/2 (Thr202/Tyr204), Akt, p-AKT (Ser473), MST1, p-MST1 (Thr183)/MST2 (Thr180), LATS1, p-LATS1 (Ser909) (Cell Signaling Technology). Antibody and antigen complexes were detected using a chemiluminescent ECL reagent (Millipore).

### Immunofluorescence staining

The cultured cells were fixed at 4 °C in ice-cold methanol for 10 min, washed three times in phosphate buffered saline (PBS), and then permeabilized in 0.1% Triton X-100/PBS for 10 min at room temperature. Nonspecific binding was blocked with 0.5% Tween-20/PBS containing 3% bovine serum albumin (BSA) for 30 min. The primary antibodies (YAP and TAZ, Cell Signaling Technology) were incubated at 4 °C overnight. The secondary antibodies (Alexa Fluor 488 goat anti-rabbit IgG, Alexa Fluor 594 goat anti-mouse IgG, Invitrogen) were incubated for 1 h at room temperature. The incubated cells were washed in PBS, and Hoechst 33342 (Gibco) was used to visualize the nuclei.

### Statistical analysis

Data are presented as mean ± SD. For data analysis, we used GraphPad Prism 6.05 software. Comparisons between two groups were analyzed via Student’s *t* test. Comparisons among three or more groups were analyzed by a one-way or two-way analysis of variance (ANOVA). Differences were considered statistically significant at *P < 0.05 and **P < 0.01.

## Results

### In vitro differentiation of adipocytes from AD-MSCs

**Fig. 1:**
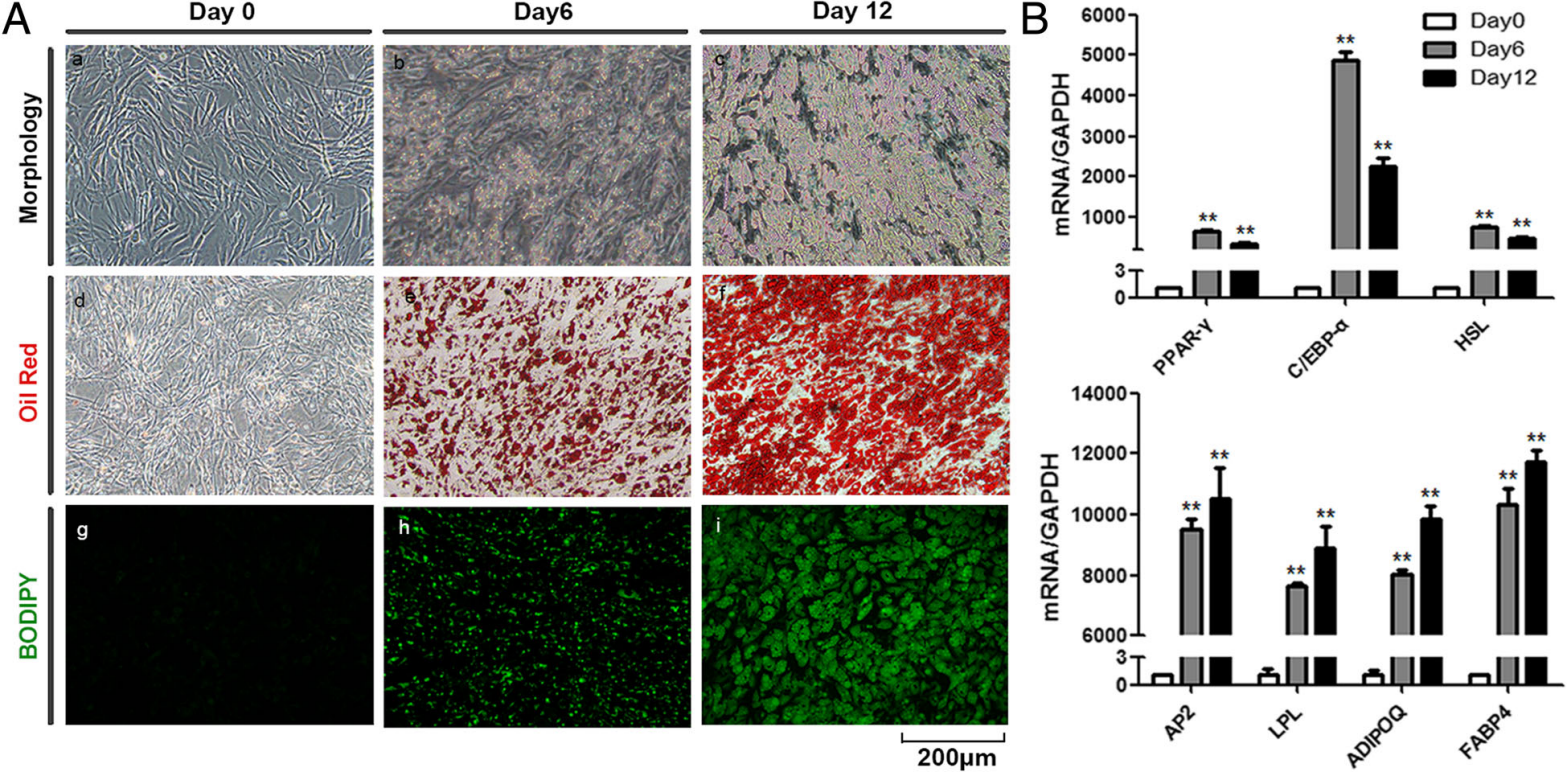
In vitro differentiation of adipocytes from AD-MSCs. **a** Morphology, Oil Red O staining, and BODIPY staining during in vitro adipocyte differentiation from human AD-MSCs. **b** Expression of specific adipogenic marker genes analyzed by qRT-PCR. GAPDH was used as internal control (**P < 0.01)

To investigate the role of adipocyte exosomes in tumor development, we first explored the feasibility of using human in vitro differentiated adipocytes as a new cellular model since most studies use mouse cell line 3T3-L1-differentiated adipocytes. hAD-MSCs were cultured under an adipogenic induction medium for 12 days, and differentiated cells exhibited typical adipocyte phenotypes as demonstrated by morphology and staining(Fig. 1a). Lipid accumulation is an important indicator of adipogenesis. The Oil Red O staining and BODIPY staining showed small round lipid droplets in differentiated adipocytes. The expression of adipocyte differentiation markers including PPARγ, c/EBPα, HSL, aP2, LPL, AdipoQ, and FABP4 was significantly increased in MSC-differentiated adipocytes as measured by qRT-PCR (Fig. 1b).

### Characterization of MSC-differentiated adipocyte exosomes

**Fig. 2:**
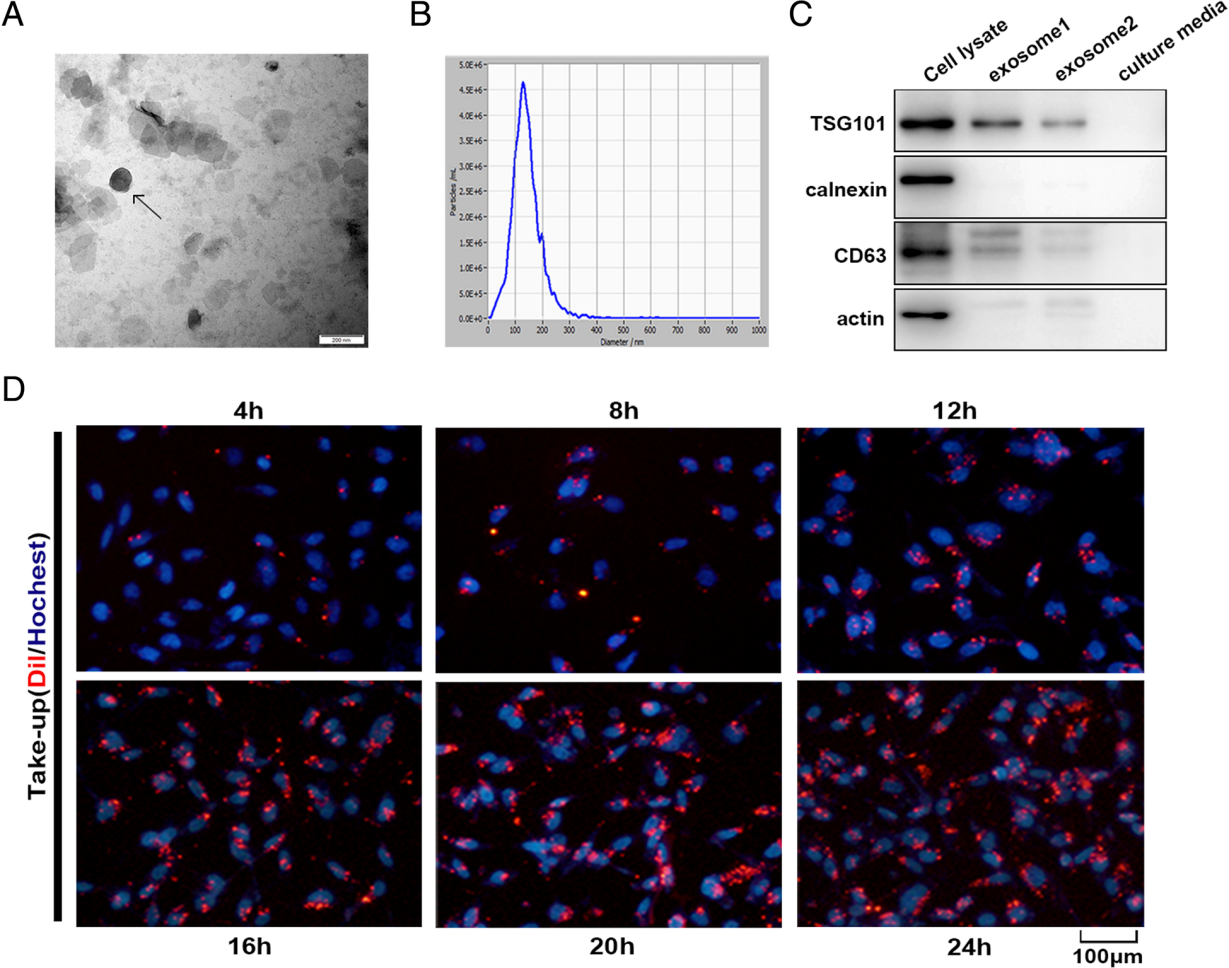
Characterization of adipocyte exosomes. **a** A representative electron microscopy image of adipocyte exosomes. Scale bar = 200 nm. **b** NTA analysis for the nanoparticle size distribution of adipocyte exosomes. **c** Western blot analysis of exosome marker CD63, TSG101, and cell-specific marker calnexin. Loaded protein for exosome 1 was 20 μg and exosome 2, 10 μg. **d** Breast cancer cells MCF7 were incubated with 200 μg/mL Dil-labeled adipocyte exosomes for the indicated times, and internalization of exosomes was determined by fluorescence microscopy. Scale bar = 100 μm

Exosomes released by MSC-differentiated adipocytes were observed under a transmission electron microscope and found to present typical exosome ultrastructure (Fig. 2a) and diameter ranging from 30 to 200 nm (Fig. 2b). Western blot showed the absence of the cell-specific marker calnexin or actin and the enrichment of the exosomal marker CD63 and TSG101 in adipocyte exosomes (Fig. 2c). Adipocyte exosomes labeled with the membrane dye Dil were readily observed under a fluorescent microscope 4 h after co-culture with breast cancer cell MCF7 and reached peak after 20–24 h (Fig. 2d). Together, we show that human in vitro differentiated adipocytes secrete exosomes with common exosomal features, which are actively taken up by breast cancer cells.

### MSC-differentiated adipocyte exosomes promote breast cancer cell proliferation and migration

**Fig. 3:**
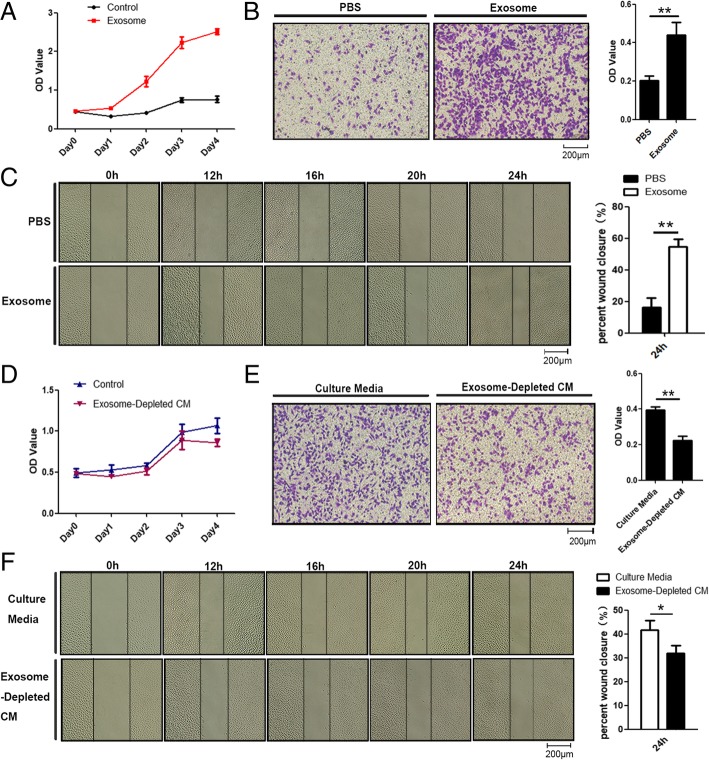
Adipocyte exosomes promote breast cancer cell proliferation and migration. **a** MTS analysis of MCF7 cells treated with adipocyte exosomes or PBS. **b** Transwell migration assays showed that MCF7 cells treated with adipocyte exosomes have a higher migratory capacity. Left is a representative microscopic image of crystal violet staining. Right shows the statistical results. **c** Migratory ability of MCF7 co-cultured with adipocyte exosomes or PBS was determined by wound healing assay. Left is a representative microscopic image. Right shows the statistical results at 24 h. **d** MTS analysis of MCF7 cells treated with a culture medium or exosome-depleted culture medium. **e** Transwell migration assays showed that MCF7 cells treated with a culture medium have higher migratory capacity than an exosome-depleted culture medium. Left is a representative microscopic image of crystal violet staining. Right shows the statistical results. **f** Migratory ability of MCF7 in a culture medium or exosome-depleted culture medium was determined by wound healing assay. Left is a representative microscopic image. Right shows the statistical results at 24 h

We then evaluated MSC-differentiated adipocyte exosomes’ effects on breast cancer cell proliferation and migration and characteristic abilities of tumor development. The proliferation rate of MCF7 cells treated with exosomes was significantly increased compared with that of control cells treated with PBS as showed by MTS assay (Fig. 3a). Both wound healing assay and transwell assay demonstrated that MCF7 cells treated with adipocyte exosomes have a higher migration rate than control cells as manifested by more numbers of migrated cells (Fig. 3b) and faster scratch wound seal (Fig. 3c). Next, we assessed whether physically removing exosomes from MSC-differentiated adipocyte-conditioned media would affect the conditioned medium’s ability to increase cell proliferation and migration. As expected, compared with the control, MCF7 cultured with the exosome-depleted adipocyte-conditioned medium have slightly lower proliferation (Fig. 3d) and migration capacity at 24 h (Fig. 3e, f).

### MSC-differentiated adipocyte exosomes reduce breast cancer cell apoptosis

**Fig. 4:**
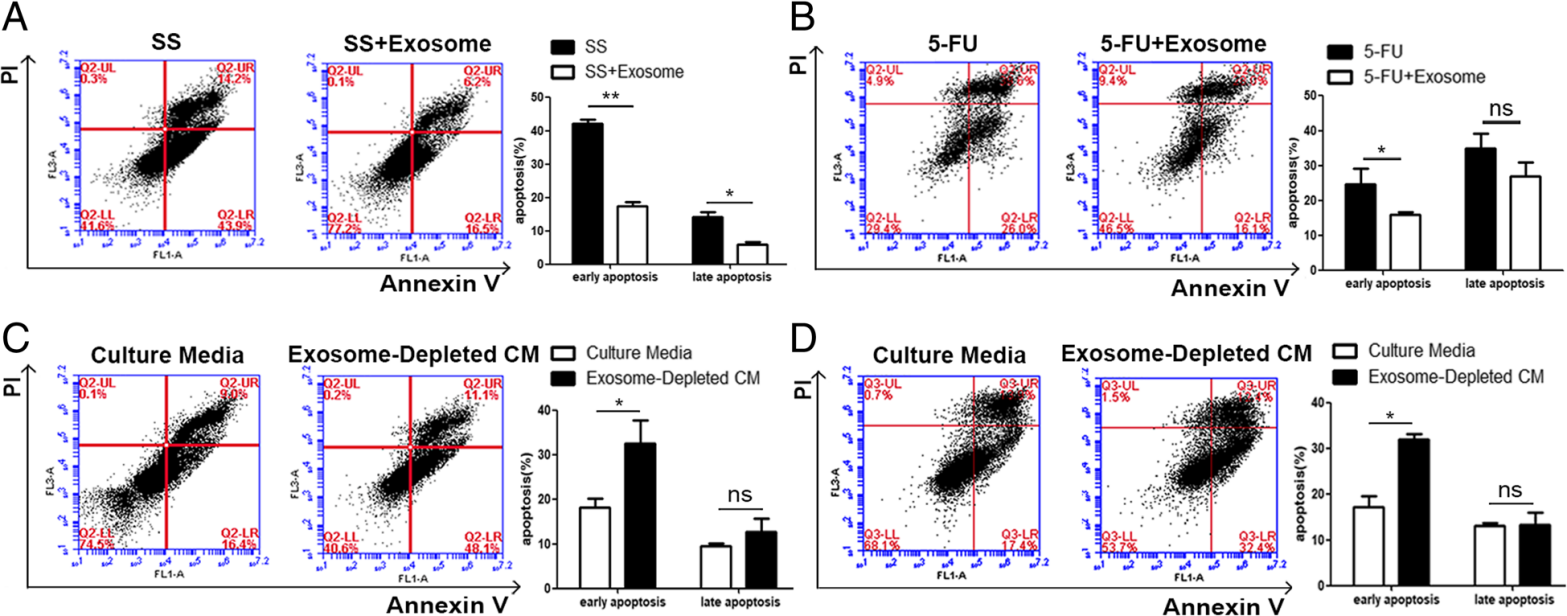
Adipocyte exosomes reduce breast cancer cell apoptosis. **a** Flow cytometric analysis of SS-induced apoptosis in MCF7 cells treated with or without adipocyte exosomes. Cells in the lower right quadrant (Annexin V+/PI−) are the early apoptotic cells, and those in the upper right quadrant (Annexin V+/PI+) represent late apoptotic cells. The experiments were performed in triplicate. The representative images are shown at the left, and the quantitative data are presented at the right. The data are presented as the mean ± SEM (*n* = 3). **b** Flow cytometric analysis of 5FU-induced apoptosis in MCF7 cells treated with or without adipocyte exosomes. The representative images are shown at the left, and the quantitative data are presented at the right. The data are presented as the mean ± SEM (*n* = 3). **c** Flow cytometric analysis of MCF7 cells cultured in an adipocyte-conditioned medium (without serum) and exosome-depleted adipocyte-conditioned medium (without serum) for 48 h. The representative images are shown at the left, and the quantitative data are presented at the right. The data are presented as the mean ± SEM (*n* = 3). **d** Flow cytometric analysis of 5FU-induced apoptosis in MCF7 cells treated with a culture medium or exosome-depleted culture medium. The representative images are shown at the left, and the quantitative data are presented at the right. The data are presented as the mean ± SEM (*n* = 3)

Another hallmark of cancer cells is their ability to thrive despite serum starvation (SS) and chemotherapeutic drug treatment. To characterize the possible effects of adipocyte exosomes on this hallmark, we added MSC-differentiated adipocyte exosomes into culture media of breast cancer cells treated with SS or chemotherapeutic drug 5FU. As showed by Annexin V/PI staining, upon treatment with SS for 48 h, early apoptotic cells (Annexin V+/PI−) and late apoptotic cells (Annexin V+/PI+) were significantly reduced in the presence of adipocyte exosomes (Fig. 4a). Similarly, adipocyte exosomes also reduced early apoptotic cells in MCF7 treated with the chemotherapeutic drug 5FU (60 μM) (Fig. 4b). Additionally, to mimic an SS condition, we cultured MCF7 in the adipocyte-conditioned medium (without serum) and exosome-depleted adipocyte-conditioned medium (without serum) for 48 h and found more apoptotic cells when exosomes were depleted (Fig. 4c). Similarly, in the presence of the chemotherapeutic drug 5FU, exosome depletion leads to more apoptotic cells (Fig. 4d). Collectively, these results suggest that adipocyte exosomes are an important participant in regulating breast cancer cell proliferation, migration, and apoptosis.

### MSC-differentiated adipocyte exosomes contribute to breast cancer growth in vivo

**Fig. 5:**
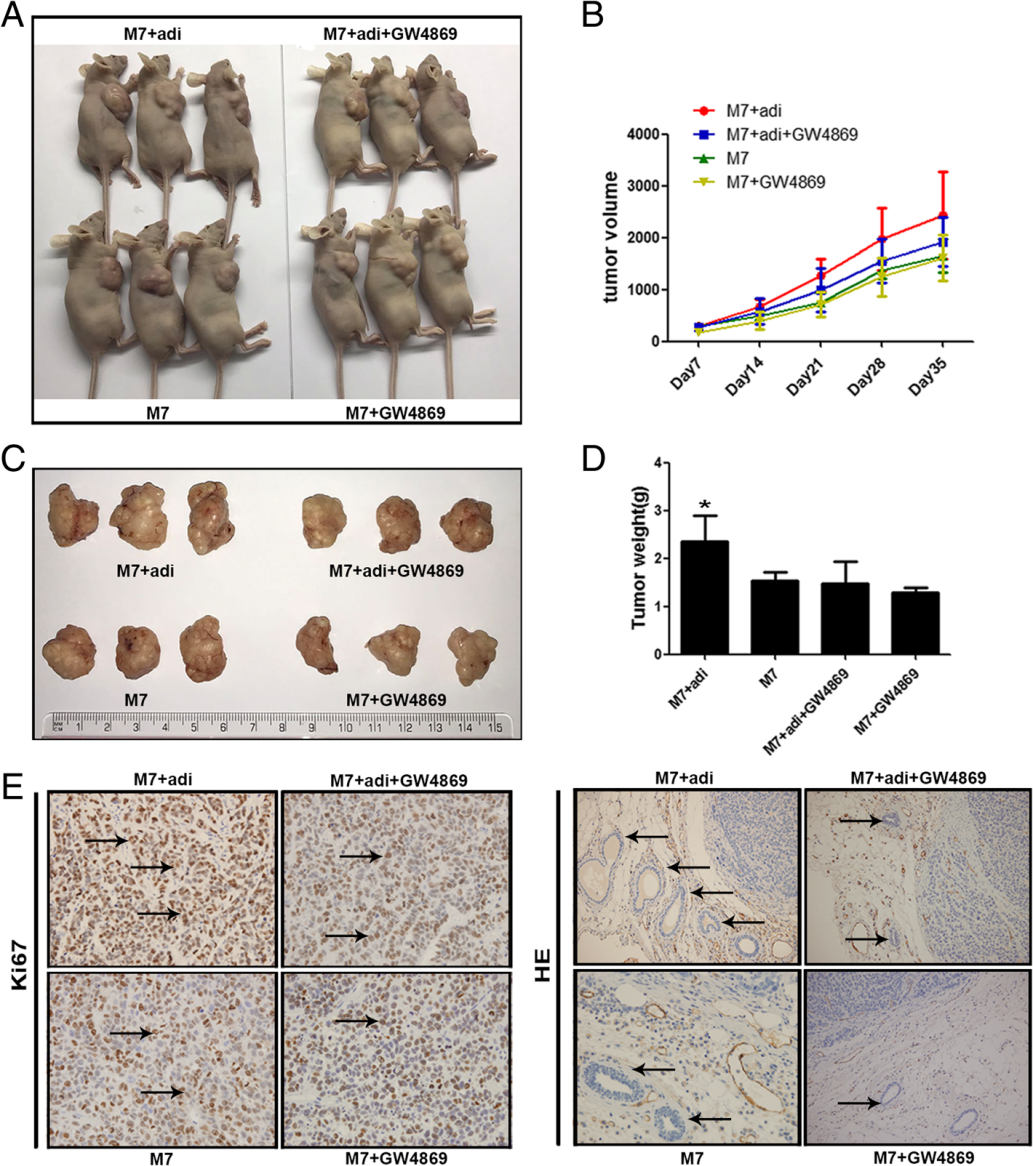
Adipocyte exosomes contribute to human MCF7 growth in vivo. **a** Tumor volume was measured and calculated every week. **b**, **c** Representative photographs of tumors generated from nude mice injected with MCF7 mixed with Matrigel alone or with human MSC-differentiated adipocytes previously treated with or without GW4869. **d** Tumor weight was measured 35 days after cell injection. **e** Reprehensive images of immunohistochemistry analysis of proliferation cells (ki-67 positive) in frozen sections from tumors. **f** Reprehensive images of HE analysis of blood vessel density in frozen sections from xenografts

To explore the contribution of adipocyte exosomes in vivo, we carried out mouse xenograft experiments by subcutaneously injecting breast cancer cells MCF7 mixed with Matrigel alone or with human MSC-differentiated adipocytes previously treated with or without GW4869, an inhibitor of exosome biogenesis/release. The presence of MSC-differentiated adipocytes showed a trend of increased tumor growth over the 35-day follow-up period while blockade of exosome generation with GW4869 seemed to reduce tumor-promoting effects of MSC-differentiated adipocytes (Fig. 5a–d). We determined the rate of cell proliferation by IHC staining of the tumor sections with the anti-Ki67 and found that the number of Ki67-positive cells was increased in the presence of MSC-differentiated adipocytes compared to the MCF7 alone group, but the increased trend was abolished when exosome generation was blocked (Fig. 5e). Similarly, the number of blood vessels was increased in the presence of adipocytes but reduced when exosome generation was blocked (Fig. 5f). Thus, these results indicate that adipocyte exosomes could contribute to tumorigenesis of breast cancer cells in vivo.

### Transcriptome analysis of breast cancer cells treated with MSC-differentiated adipocyte exosomes

**Fig. 6:**
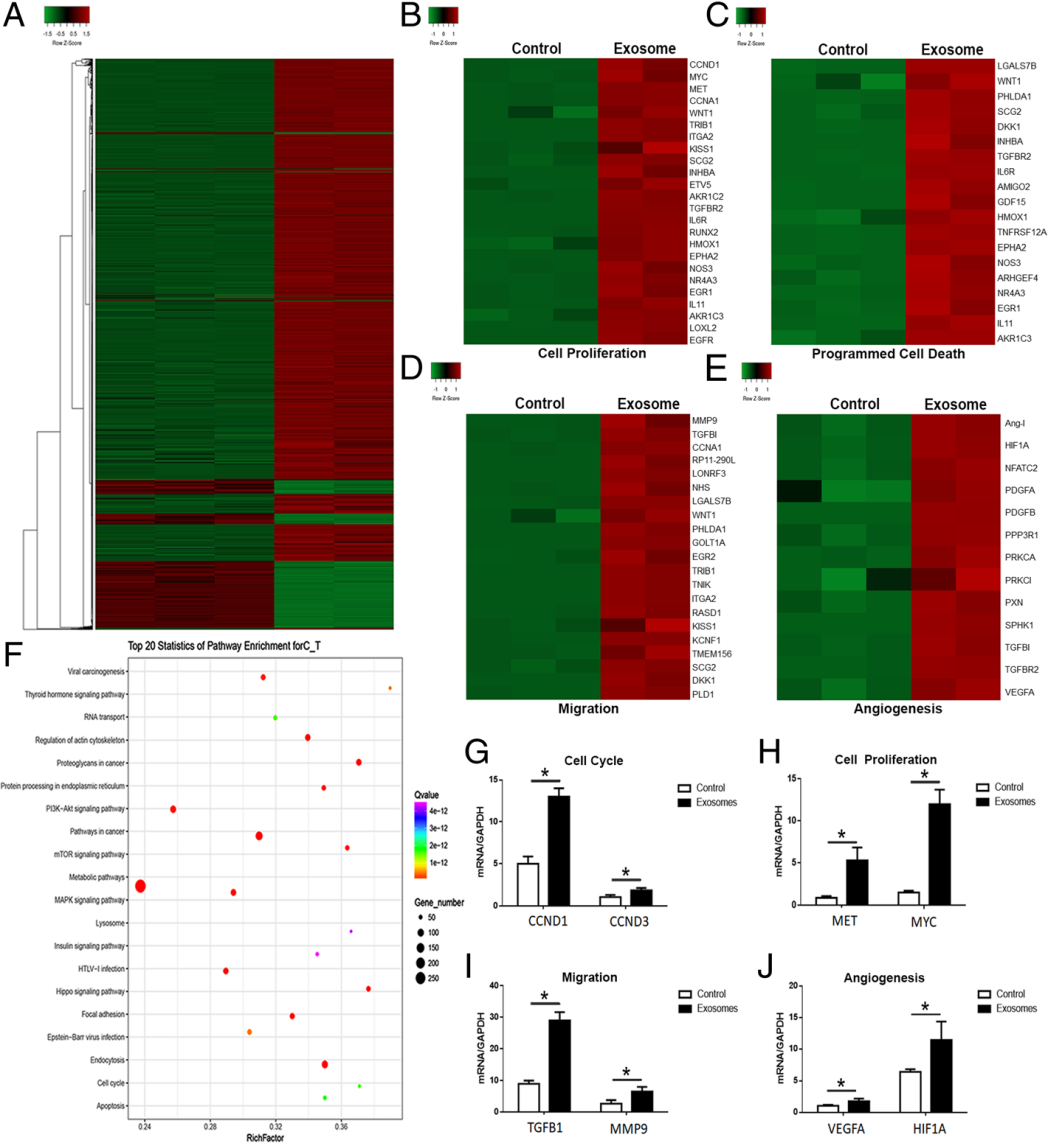
Transcriptome analysis of breast cancer cells treated with adipocyte exosomes. **a** Heat map showed differentially expressed genes in adipocyte exosome-treated MCF7 and control MCF7. **b**–**e** Upregulation of cancer phenotype-associated genes in adipocyte exosome-treated MCF7. **b** Genes associated with cell proliferation. **c** Genes associated with programmed cell death. **d** Genes associated with migration. **e** Genes associated with angiogenesis. **f** Top 20 signaling pathways enriched in adipocyte exosome-treated MCF7. **g**–**j** qRT-PCR confirmed the increased expression of selected genes associated with cell cycle (G, CCND1, CCND3), cell proliferation (**h**, MET, MYC), migration (**i**, TGFB1, MMP9), and angiogenesis (**j**, VEGFA, HIF1A)

We next evaluated the transcriptomic alterations induced by adipocyte exosomes and identified activated signaling pathways in MCF7. Adipocyte exosomes could convert MCF7 into a transcriptional active state as demonstrated by more upregulated genes (Fig. 6a). Unsupervised clustering identified upregulation of gene signatures related to cell proliferation, programmed cell death, migration, and angiogenesis in adipocyte exosome-treated MCF7 (Fig. 6b–e). qRT-PCR analysis confirmed the increased expression of selected genes from the abovementioned gene signatures (Fig. 6g–j). Interestingly, KEGG analysis identified at least 20 signaling pathways with known functions in tumor development (Fig. 6f).

### MSC-differentiated adipocyte exosomes activated the Hippo signaling pathway in breast cancer cells

**Fig. 7:**
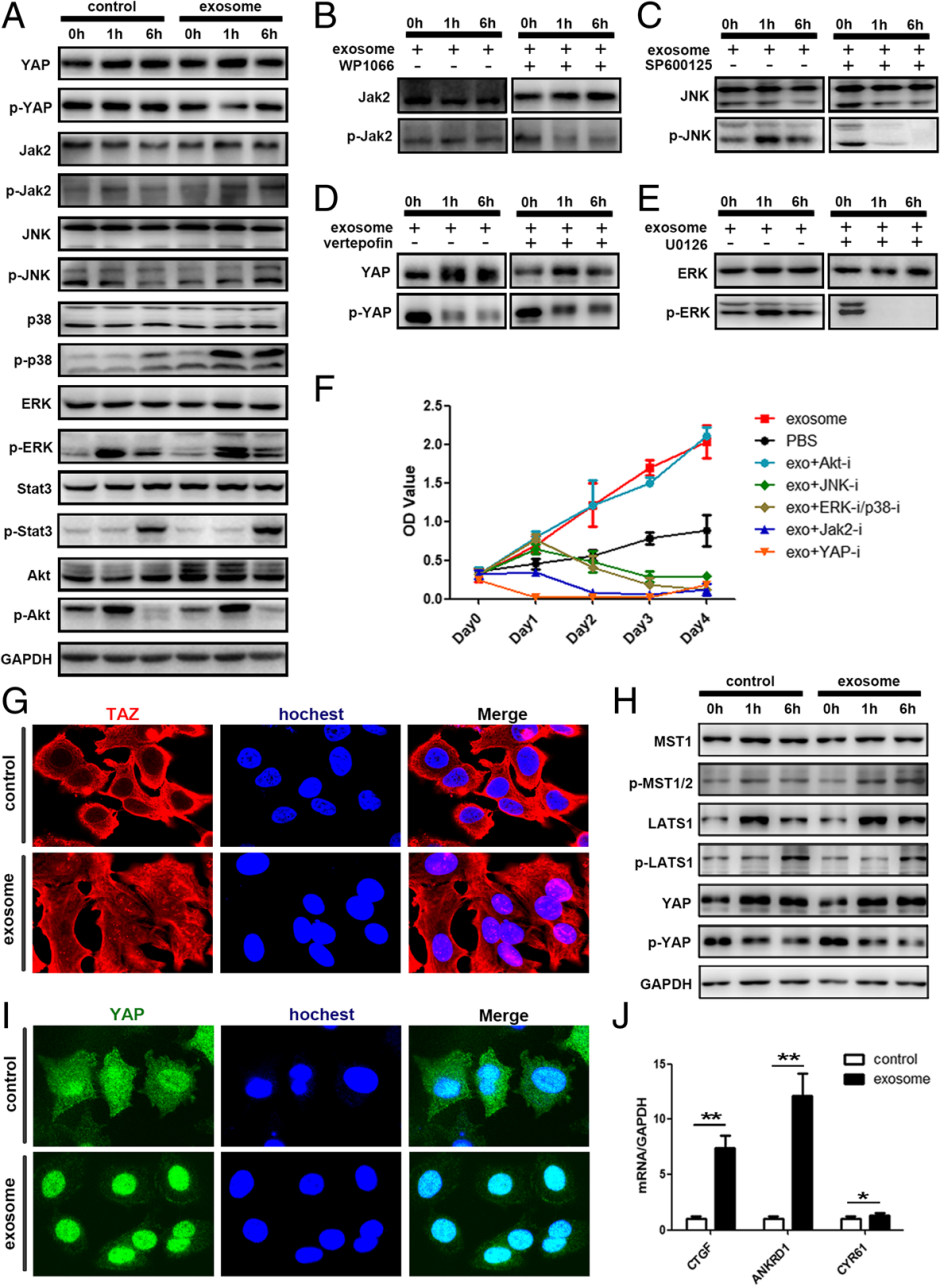
Adipocyte exosomes activated the Hippo signaling pathway in MCF7 cells. **a** Phosphorylation of YAP, Jak2, JNK, P38, ERK, Stat3, and AKT was analyzed by western blot. GAPDH was used as the loading control. **b**–**e** Phosphorylation of key proteins was analyzed after addition of specific signaling pathway inhibitor. **b** WP1066 inhibitor of JAK2/STAT3. **c** SP600125 inhibitor of JNK. **d** Verteporfin inhibitor of Hippo. **e** U0126 inhibitor of ERK/P38. **f** MTS analysis of MCF7 cells treated with adipocyte exosomes or PBS in the presence or absence of different signaling pathway inhibitors. **g** Representative immunofluorescence staining images of nuclear translocation of TAZ in adipocyte exosome-treated MCF7. **h** Phosphorylation of key proteins of the Hippo signaling pathway. **i** Representative immunofluorescence staining images of nuclear translocation of YAP in adipocyte exosome-treated MCF7. **j** Expression of YAP/TAZ downstream genes. GAPDH was used as an internal control

Among them, we chose PI3K-Akt, MAPK, Hippo, and JAK-STAT for further analysis. Western blot confirmed the phosphorylation of JAK, JNK, ERK, and P38 as well as the dephosphorylation of YAP (Fig. 7a). The corresponding pathway inhibitors altered such a phosphorylation status (Fig. 7b–e) and attenuated the tumor growth-promoting effect of adipocyte exosomes, with Hippo inhibition exhibiting the most significant effect (Fig. 7f). Specifically, Fig. 4h demonstrates the increased phosphorylation of YAP upstream kinase MST1. Figure 7g, i shows the transportation of YAP/TAZ (two key transcription factors of the Hippo signaling pathway) into the nucleus where they activate transcription of the downstream genes such as CTGF, ANKDR1, and CYR61(Fig. 7j). Collectively, these results showed that adipocyte exosomes activated the Hippo signaling pathway in the MCF7 cells.

## Discussion

Numerous studies have demonstrated that the tumor microenvironment could cooperate to modulate malignant behaviors of tumors cells [5, 28, 29]. In breast cancer, the cellular components of tumor microenvironments include resident fibroblasts, adipocytes, a number of recruited immune cells, and newly formed blood vessels with their associated cells [30]. Dynamic and reciprocal communication between tumor cells and surrounding compartments has been intensively investigated. However, in this context, very little attention has been given to adipocytes, although they represent the most prominent cell type in breast tumor microenvironment. Traditionally, adipocytes are thought to function as energy storage cells. Now, accumulating evidence suggests that they could also serve as endocrine cells by secreting adipokines [31]. Here, we chose MSC-differentiated adipocytes as a cellular model to study interactions between adipocytes and breast cancer cells. The adipocytes are differentiated by culturing human MSCs under adipogenic conditions and are fully characterized by morphology, staining, and marker gene expression. We found the tumor-promoting effects of MSC-differentiated adipocytes were reduced when adipocyte exosomes were depleted.

In recent years, much interest has been devoted to exosomes, which function as carriers of bioactive proteins, lipids, and nuclear acids and are increasingly regarded as crucial players in cell-cell communications [32, 33]. In the present study, we characterized exosomes secreted by in vitro differentiated human adipocytes. The uptake of these exosomes by breast cancer cells MCF7 was observed by immunofluorescence staining, confirming direct interaction between adipocyte exosomes and cancer cells. Adipocyte exosome treatment brings about sustained changes in the proliferation and anti-apoptosis of breast cancer cells. To our knowledge, this is the first reported about the effects of MSC-differentiated adipocyte exosomes on breast cancer cells. Previous studies have explored the role of adipocyte exosomes in inflammation and insulin resistance. For example, adipocyte exosomes could differentiate monocytes into macrophages characteristic of human adipose tissue macrophages (ATM), defined by release of both pro- and anti-inflammatory cytokines [34]. Wang et al. found adipocyte exosomes could aggravate atherosclerosis by increasing vasa vasorum angiogenesis in diabetic ApoE−/− mice [15] while adipocyte exosomes induce insulin resistance in skeletal muscle [16]. Adipocyte exosomes have also been reported to promote the growth of hepatocellular carcinoma by targeting deubiquitination-related USP7 [35] and promote melanoma aggressiveness through fatty acid oxidation [36]. Here, our study added a new function of adipocyte exosomes in breast cancer regulation. Currently, it remains largely unknown how adipocytes influence breast tumor cell behavior or whether any of the paracrine factors secreted by adipocytes cause changes in the phenotypic behavior of the malignant cells. Our results provide new insights into exosomes which are emerging as a novel way of cell-cell communication.

Hippo signaling is one of the major pathways controlling tumorigenesis. Key components of the Hippo pathway regulate breast tumor growth, metastasis, and drug resistance [37]. Here, we found adipocyte exosomes could activate two key downstream effector proteins of Hippo, the YAP and TAZ, as demonstrated by Fig. 7.

Taken together, we found that in vitro hAD-MSC-differentiated adipocytes could secrete exosomes which are actively taken up by breast cancer cell line MCF7 and subsequently promote breast cancer cell MCF7 proliferation and migration as well as protect MCF7 from serum derivation or chemotherapeutic drug-induced apoptosis in vitro. In the in vivo mouse xenograft model, depletion of exosomes reduced tumor-promoting effects of adipocytes which implies the contribution of adipocyte exosomes in the tumor microenvironment. Furthermore, we found the Hippo signaling pathway was partially responsible for the tumor-promoting effects of adipocyte exosomes. Our data suggest that adipocyte exosomes could act as an additional mechanism contributing to breast tumor microenvironment and may offer a novel therapeutic modality to target breast cancer growth. However, our study has a limitation. The in vivo mouse xenograft experiment was performed using adipocytes treated with GW4869 while our in vitro studies were done with pure exosomes. More studies are needed to further explore the in vivo role of adipocyte exosomes.

## Conclusions

Collectively, our data indicated that (i) adipocyte exosomes could be actively incorporated into breast cancer cells and significantly changed transcriptome, particularly genes associated with tumor development, (ii) depletion of exosomes from adipocyte reduced tumor-promoting effects of adipocytes, and (iii) the Hippo signaling pathway was activated in adipocyte exosomes which treated breast cancer cells. Our results provided new insights into the role of adipocyte exosomes in the breast tumor microenvironment.

## Abbreviations

FBS: Fetal bovine serum
FFAs: Free fatty acids
H-DMEM: High glucose of Dulbecco’s modified Eagle’s medium
HE: Hematoxylin-eosin
MSCs: Mesenchymal stromal/stem cells
PVDF: Polyvinylidene difluoride
qRT-PCR: Quantitative real-time PCR
